# Association Between Low-Dose Ethambutol Therapy and Optic Neuropathy in *Mycobacterium avium* Complex Pulmonary Disease: A Retrospective Cohort Study Using Propensity Score Analysis

**DOI:** 10.1093/ofid/ofag127

**Published:** 2026-03-03

**Authors:** Yasuhiro Morimoto, Hiroki Matsui, Masaki Ikeda, Norihiko Kubota, Tatsuya Nagai, Ayumu Otsuki, Hiroyuki Ito, Kei Nakashima

**Affiliations:** Department of Pulmonology, Kameda Medical Center, Kamogawa, Chiba, Japan; Division of Respiratory Diseases, Department of Internal Medicine, The Jikei University School of Medicine, Tokyo, Japan; Department of Clinical Epidemiology and Health Economics, School of Public Health, the University of Tokyo, Tokyo, Japan; Clinical Research Support Office, Kameda Medical Center, Kamogawa, Chiba, Japan; Department of Pulmonology, Kameda Medical Center, Kamogawa, Chiba, Japan; Department of Pulmonology, Kameda Medical Center, Kamogawa, Chiba, Japan; Department of Pulmonology, Kameda Medical Center, Kamogawa, Chiba, Japan; Department of Pulmonology, Kameda Medical Center, Kamogawa, Chiba, Japan; Department of Pulmonology, Kameda Medical Center, Kamogawa, Chiba, Japan; Department of Pulmonology, Kameda Medical Center, Kamogawa, Chiba, Japan

**Keywords:** ethambutol, mycobacterium avium complex, nontuberculous mycobacteria, optic neuropathy

## Abstract

**Background:**

Current guidelines recommend ethambutol as a first-line agent for treating *Mycobacterium avium* complex pulmonary disease (MAC-PD) but can induce optic neuropathy. Previous studies suggest that low-dose ethambutol may reduce the incidence of optic neuropathy without compromising its effectiveness, but have not adequately adjusted for risk factors for optic neuropathy. We investigated the association between low-dose ethambutol therapy and optic neuropathy in patients with MAC-PD.

**Methods:**

This retrospective cohort study included patients treated for MAC-PD at Kameda Medical Center between April 2003 and July 2024. Patients were categorized into low-dose (<12.5 mg/kg/day) and high-dose (≥12.5 mg/kg/day) ethambutol groups. The primary outcome was the incidence of optic neuropathy. Secondary outcomes included failure of negative culture conversion and macrolide resistance. Propensity score-based overlap weighting was used to adjust for patient characteristics and outcomes were compared using datasets generated through bootstrap resampling.

**Results:**

Of 223 patients, 79 received low-dose (10.7 mg/kg/day) and 144 received high-dose ethambutol (15.4 mg/kg/day). None of the patients in the low-dose group and 4.8% in the high-dose group developed optic neuropathy. After adjustment, the risk of optic neuropathy was significantly lower in the low-dose ethambutol group (risk difference: −17.1%, 95% CI: −32.9% to −5.4%), but the risk difference for failure of negative culture conversion and macrolide resistance did not differ significantly between groups (−20.0%, 95% CI: −45.6% to 2.5%; and −4.9%, 95% CI: −13.5% to 0.0%, respectively).

**Conclusions:**

Low-dose ethambutol therapy may reduce the risk of optic neuropathy without compromising treatment outcomes, offering a safer option for MAC-PD.

## Teaser

Optic neuropathy incidence was significantly lower in the low-dose than the high-dose ethambutol group, whereas incidence of failure of negative culture conversion and macrolide resistance did not differ significantly between groups, demonstrating that low-dose ethambutol reduces toxicity without compromising effectiveness.

The incidence and prevalence of nontuberculous mycobacterial pulmonary disease (NTM-PD) is increasing worldwide, with *Mycobacterium avium* complex (MAC) being the most common causative species globally and in Japan [1–5]. International guidelines recommend multidrug regimens including macrolides, ethambutol, and rifampin for treating MAC pulmonary disease (MAC-PD) [1]. Ethambutol plays a critical role in preventing macrolide resistance [6, 7] and improving treatment outcomes, such as sputum culture conversion and microbiological cure [8]. Using a fluoroquinolone as a substitute for ethambutol is associated with higher treatment failure rates [9]. However, multidrug treatment for MAC-PD requires long-term administration which is often associated with adverse treatment-related events [10, 11]. Despite its clinical effectiveness, ethambutol is frequently discontinued owing to the development of optic neuropathy. Once optic neuropathy has been confirmed by ophthalmological evaluation, permanent discontinuation of ethambutol is recommended [2]. Consequently, optimizing ethambutol administration to reduce the risk of optic neuropathy while maintaining the effectiveness of treatment remains a critical clinical challenge.

Two recent single-center retrospective cohort studies from Japan have evaluated the use of low-dose ethambutol therapy [12, 13]. Both studies classified patients into a low-dose group and a high-dose group, using 12.5 mg/kg/day as the cutoff. Ando et al [12]. reported that low-dose ethambutol may reduce ocular toxicity without increasing the risk of failure of negative sputum culture conversion or radiological deterioration. Despite the higher incidence of optic neuropathy in the high-dose group, treatment outcomes such as culture conversion, radiological improvement, and macrolide resistance did not differ significantly between groups. Similarly, Watanabe et al [13]. reported that an ethambutol dose of ≤12.5 mg/kg/day in guideline-based chemotherapy may reduce the incidence of optic neuropathy without compromising clinical outcomes. In their study, no macrolide resistance was observed, and the rates of negative culture conversion and microbiological cure did not differ significantly between the low-dose and high-dose groups. Although the incidence of optic neuropathy was higher in the high-dose group (7.1% vs 1.0%), this difference was not statistically significant (*P* = .07). Taken together, these findings suggest that low-dose ethambutol therapy may offer a favorable balance between effectiveness and safety, but larger studies with appropriate adjustment for clinical confounders are warranted. Although risk factors for ethambutol-induced optic neuropathy, such as age, kidney dysfunction, and comorbidities, have been reported [14–16], to our knowledge, no prior studies have adequately adjusted for these clinical confounders. Therefore, further investigations based on real-world clinical practice data with appropriate adjustments for baseline characteristics are needed.

In this single-center cohort study, we aimed to evaluate the association between low-dose ethambutol therapy and optic neuropathy in patients with MAC-PD after adjusting for patient characteristics.

## METHODS

### Study Setting and Population

We conducted a single-center, retrospective, observational cohort study at Kameda Medical Center, a tertiary care hospital in Japan. The records of patients diagnosed with MAC-PD who received guideline-based daily treatment between April 2003 and July 2024, were reviewed retrospectively. Diagnosis criteria and guideline-based treatment were based on the American Thoracic Society/European Respiratory Society/European Society of Clinical Microbiology and Infectious Diseases/Infectious Diseases Society of Americaclinical practice guidelines [1]. The index date was defined as the date of initiating multidrug treatment. The exclusion criteria were: (1) prior treatment history for NTM-PD; (2) not receiving a multidrug regimen including ethambutol; (3) missing body weight data required for calculating the ethambutol dose (mg/kg/day); (4) administration of intermittent therapy, as this study specifically focused on daily regimens; and (5) loss to follow-up immediately after the index date.

The study protocol was approved by the Research Ethics Committees of Kameda Medical Center (#24-047). Because of the retrospective design and use of anonymized patient information, the requirement for written informed consent was waived.

### Treatment

All patients included in this study received daily combination therapy with at least 3 drugs, including macrolides and ethambutol, in accordance with international guidelines [1]. These guidelines generally recommend a standard ethambutol dose of 15 mg/kg/day [1]. The initial dose of ethambutol was determined at the discretion of the treating physician based on each patient's clinical background. Macrolide therapy consisted of clarithromycin or azithromycin. Because azithromycin was not approved for MAC-PD in Japan until 2020, most patients received clarithromycin in accordance with a domestic position paper. Treatment was generally continued for at least 1 year after culture conversion, with dosage and duration adjusted according to the patient's clinical course. Patients were followed up during treatment or until loss to follow-up. Adverse events were recorded by the physician, and treatment was discontinued or terminated as clinically indicated. An ophthalmologist performed a baseline ophthalmological evaluation before the initiation of ethambutol therapy and patients underwent regular ophthalmological checkups during treatment to monitor for early signs of optic neuropathy. In addition, the attending physician promptly referred patients reporting any visual symptoms to the ophthalmologist for an unscheduled assessment, regardless of the routine follow-up schedule. When ethambutol-associated optic neuropathy was diagnosed based on an ophthalmological evaluation, the administration of ethambutol was permanently discontinued. If the visual symptoms were assessed as being unrelated to ethambutol, ethambutol administration was continued, as appropriate.

During the study period, a nationwide temporary shortage of ethambutol occurred in Japan from March to May 2023 due to production-line issues. During this time, some patients at our facility temporarily received a newer-generation fluoroquinolone as a substitute for ethambutol. These cases were carefully managed, and ethambutol therapy was resumed once the supply was restored.

### Exposure According to Ethambutol Dose

Patients were divided into 2 groups based on their initial ethambutol dose: a low-dose group and a high-dose group. The low-dose group was defined as patients who received a daily ethambutol dose of <12.5 mg/kg/day, and the high-dose group was defined as those as those who received a daily ethambutol dose of ≥12.5 mg/kg/day.

### Outcomes

The primary endpoint was the incidence of ethambutol-induced optic neuropathy. Based on previous studies [12, 17], ethambutol-induced optic neuropathy was defined as: (1) a change in visual acuity and/or color vision discrimination, observed both subjectively and objectively, that could not be explained by any other condition and that improved and/or returned to baseline or progressed after discontinuation of ethambutol; and (2) diagnosis by ophthalmologists based on assessment including visual acuity, visual field testing, fundus examination, color vision testing, and central flicker testing.

The secondary endpoints were the proportion of patients with failure of negative culture conversion and the incidence of macrolide resistance. The standard indicator of treatment outcome for NTM-PD is considered to be negative culture conversion and microbiological cure [18]. However, many patients were unable to expectorate sputum. Based on previous studies [13, 19, 20], we considered a method of regarding cases who could not expectorate sputum during treatment as achieving negative culture conversion, but we decided that this could lead to overestimation of the effectiveness of treatment. Therefore, we set the failure of negative culture conversion as an outcome indicator, consistent with the use of this indicator in a previous study [12]. This was defined as the persistence of positive cultures in patients who were able to provide sputum samples.

### Data Collection, Microbiological Examination, and Radiologic Evaluation

We retrospectively collected clinical, microbiological, and radiological data on each patient from their medical records. The variables included: age, sex, weight, height, smoking status (nonsmoker, or current/past smoker), underlying diseases, history of previous tuberculosis treatment, laboratory results (serum albumin, creatinine, and C-reactive protein levels, and creatinine clearance), mycobacterial species (*Mycobacterium avium* or *Mycobacterium intracellulare*), sputum smear status at treatment initiation, radiological disease type at treatment initiation, drug treatment (dose and duration), incidence of optic neuropathy, sputum culture results, and development of macrolide resistance after treatment initiation.

For microbiological testing, acid-fast bacillus (AFB) smears were performed using either the Ziehl-Neelsen staining technique or the fluorescent staining method. Acid-fast bacillus cultures were conducted using both Ogawa medium and the BD BACTEC MGIT 960 system (MGIT; Becton Dickinson, Franklin Lakes, NJ, USA). Drug susceptibility testing was performed using a commercial panel (Broth MIC NTM or Broth MIC SGM; Kyokuto Pharmaceutical Industry Co., Ltd., Tokyo, Japan), with macrolide resistance defined as a minimum inhibitory concentration (MIC) of ≥32 μg/mL for clarithromycin.

Radiological findings from chest computed tomography were evaluated by 2 pulmonologists and used to classify the disease into one of four radiological types [21]: non-cavitary nodular bronchiectatic (NC-NB), C-NB, fibrocavitary (FC), and unclassifiable (UC).

### Statistical Analyses

Descriptive statistics for baseline characteristics and outcomes were collected for each group. Comparisons between groups were performed using independent-samples t-tests for continuous variables and χ-square tests for categorical variables. Because this was a retrospective cohort study, we did not perform prior sample size calculations, and all patients who met the eligibility criteria were included in the analysis.

Because several variables had missing values, we performed multiple imputation by chained equation to generate 50 imputed datasets through 100 iterations [22]. For each imputed dataset, 200 bootstrap resamples were created (for a total of 10 000 bootstrap-imputed samples). Outcome proportions in the low- and high-dose groups were estimated in each bootstrap-imputed sample, and risk differences were calculated as the difference between groups (low-dose group minus the high-dose group). The distribution of these risk differences across all samples was used to determine the median and 95% CIs (2.5th to 97.5th percentiles) of the risk difference.

To reduce confounding, propensity scores were calculated for low-dose ethambutol and overlap weighting was performed using these propensity scores [23]. To calculate propensity scores, a logistic regression model was constructed with low-dose ethambutol as the dependent variable and age, sex, body mass index (BMI), creatinine clearance, presence of ophthalmic disorders, presence of hypertension, *M. intracellulare*, radiological disease type (NC-NB, C-NB, FC, or UC), and positive AFB smear as the explanatory variables. We selected these explanatory variables considering risk factors for ethambutol-related optic neuropathy [14–16], prognostic factors for NTM-PD [24, 25], and ophthalmic disease complications [12]. The standardized mean difference (SMD) was used to assess covariate balance between the groups. According to previous studies [26], the groups were considered balanced when the SMD was <0.1.

All statistical analyses were performed using R software (version 4.3.0; R Development Core Team). Two-tailed *P* values < .05 were considered statistically significant.

### Sensitivity Analysis

To evaluate the robustness of our findings, we performed a sensitivity analysis restricted to patients treated with ethambutol for 5 years or less. This analysis was conducted to minimize the potential influence of treatment duration. We applied the same statistical methodology as in the primary analysis, using propensity score-based overlap weighting and the bootstrap method.

## RESULTS

**Figure 1.**
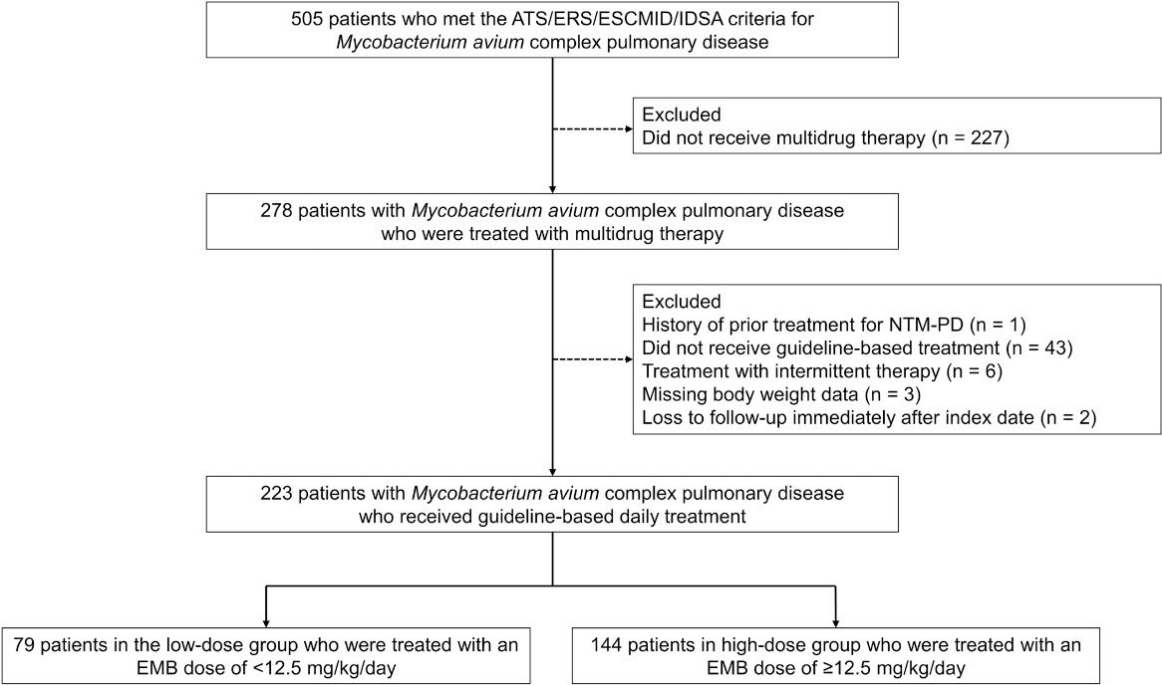
Study flowchart and patient selection. Abbreviations: ATS, American Thoracic Society; EMB, ethambutol; ERS, European Respiratory Society; ESCMID, European Society of Clinical Microbiology and Infectious Diseases; IDSA, Infectious Diseases Society of America; NTM-PD, nontuberculous mycobacterial pulmonary disease.

A study flow chart of patient selection is shown in Figure 1. We retrospectively collected data from 505 patients diagnosed with MAC-PD who visited Kameda Medical Center. Of the 505 patients, 278 were treated with multidrug therapy. Of the 278 patients, 55 were excluded: 1 with a history of prior treatment, 43 who did not receive guideline-concordant treatment, 6 who received intermittent therapy, 3 with missing details on body weight, and 2 patients who were lost to follow-up immediately after index date. Therefore, 223 patients were included in the analysis, of whom 79 were in the low-dose group, and 144 were in the high-dose group.

Table 1 shows unadjusted and adjusted baseline clinical characteristics before imputation. Before adjustment, the daily dose of ethambutol per kg body weight differed significantly between groups (10.73 mg/kg in the low-dose group and 15.42 mg/kg in the high-dose group; SMD = 2.199). Age, female sex, BMI, ophthalmic disorders, species (*M. avium* and *M. intracellulare*), disease of radiological findings, serum creatinine levels, and creatinine clearance were imbalanced between the 2 groups (SMD >0.1). Several explanatory variables had missing values, including BMI (10.7%) and radiological disease type (9.8%). In the adjusted cohort, the daily dose of ethambutol remained significantly lower in the low-dose group. The adjustment variables, including age, female sex, BMI, ophthalmic disorders, hypertension, *M. intracellulare*, positive AFB smear, radiological disease type, and creatinine clearance were well balanced, with SMD values <0.1. Furthermore, other variables such as presence of diabetes mellitus, *M. avium*, and serum creatinine levels, were also well balanced, showing SMD values below 0.1.

**Table 1.** Baseline Clinical Characteristics of the Unadjusted and Adjusted Cohort Before Imputation

Variables	Unadjusted Cohort				Adjusted Cohort		
	Low-Dose EMB^a^ n = 79	High-Dose EMB^b^ n = 144	SMD	Missing (%)	Low-Dose EMB^a^ n = 40.43	High-Dose EMB^b^ n = 40.43	SMD
EMB dose (mg/kg/d)	10.73 (± 1.34)	15.42 (± 2.70)	2.199	0.9	10.78 (± 1.34)	14.85 (± 2.14)	2.281
EMB dose (mg/d)	542 (± 102)	704 (± 134)	1.354	0.0	530 (± 88)	715 (± 125)	1.703
Duration of EMB administration (d)	534.3 (± 405.5)	642.2 (± 655.6)	0.198	0.4	547.4 (± 403.3)	696.9 (± 706.4)	0.260
Age (y)	70.4 (± 8.9)	68.8 (± 9.1)	0.187	0.0	70.0 (± 8.9)	70.0 (± 8.6)	< 0.001
Female	65 (82.3)	106 (73.6)	0.210	0.0	31.9 (79.0)	31.9 (79.0)	< 0.001
BMI (kg/m^2^)	20.7 (± 3.5)	18.7 (± 3.0)	0.614	10.7	19.9 (± 2.9)	19.9 (± 2.9)	< 0.001
Smoking history	18 (23.1)	36 (25.9)	0.066	2.7	9.8 (24.8)	7.6 (19.5)	0.126
Previous history of TB treatment	4 (5.1)	3 (2.1)	0.161	0.0	1.8 (4.4)	1.0 (2.4)	0.111
Underlying disease	…	…	…	…	…	…	…
Ophthalmic disorders	9 (11.4)	23 (16.0)	0.134	0.0	5.9 (14.6)	5.9 (14.6)	< 0.001
Hypertension	22 (27.8)	35 (24.6)	0.073	0.9	9.8 (24.3)	9.8 (24.3)	< 0.001
Diabetes mellitus	8 (10.1)	17 (11.8)	0.054	0.0	3.8 (9.4)	4.5 (11.1)	0.054
COPD	5 (6.3)	8 (5.6)	0.033	0.0	3.0 (7.3)	1.5 (3.7)	0.160
Bronchial asthma	2 (2.5)	3 (2.1)	0.028	0.9	0.9 (2.2)	1.7 (4.1)	0.112
Interstitial pneumonia	2 (2.5)	13 (9.2)	0.285	0.9	0.7 (1.8)	3.7 (9.1)	0.329
Aspergillosis	1 (1.3)	4 (2.8)	0.110	0.9	0.8 (2.0)	1.5 (3.6)	0.100
Lung cancer	2 (2.6)	5 (3.5)	0.056	1.3	0.7 (1.8)	1.1 (2.7)	0.059
Species	…	…	…	…	…	…	…
*M. avium*	53 (67.1)	70 (50.0)	0.352	1.8	24.9 (61.6)	23.1 (57.1)	0.091
*M. intracellulare*	31 (39.2)	76 (54.3)	0.305	1.8	18.5 (45.9)	18.5 (45.9)	< 0.001
Positive AFB smear	33 (41.8)	51 (37.0)	0.099	2.7	16.1 (39.9)	16.1 (39.9)	< 0.001
Radiological disease type	…	…	0.374	9.8	…	…	< 0.001
NC-NB form	47 (60.3)	82 (66.7)	…	…	26.3 (66.6)	26.3 (66.6)	…
C-NB form	22 (28.2)	22 (17.9)	…	…	9.0 (22.8)	9.0 (22.8)	…
FC form	6 (7.7)	18 (14.6)	…	…	3.5 (8.8)	3.5 (8.8)	…
UC form	3 (3.8)	1 (0.8)	…	…	0.7 (1.8)	0.7 (1.8)	…
Blood biochemistry	…	…	…	…	…	…	…
Albumin (g/dL)	3.96 (±0.52)	3.77 (±0.64)	0.327	5.8	3.97 (±0.46)	3.79 (±0.67)	0.318
Creatinine (mg/dL)	0.64 (±0.14)	0.71 (±0.48)	0.205	0.0	0.65 (±0.14)	0.65 (±0.19)	0.013
Creatinine clearance (mL/min)	70.41 (±22.13)	62.83 (±20.69)	0.354	0.9	67.93 (±18.09)	67.93 (±22.15)	< 0.001
C-reactive protein (mg/dL)	0.40 (±0.68)	1.11 (±2.49)	0.391	5.3	0.45 (±0.76)	0.94 (±2.38)	0.274
Drug use	…	…	…	…	…	…	…
Macrolide	79 (100.0)	144 (100.0)	…	0.0	40.4 (100.0)	40.4 (100.0)	…
Clarithromycin	38 (48.1)	121 (84.0)	…	0.0	20.3 (50.2)	31.6 (78.2)	…
Azithromycin	41 (51.9)	23 (16.0)	0.820	0.0	20.1 (49.8)	8.8 (21.8)	0.610
Rifampicin	78 (98.7)	144 (100.0)	0.160	0.0	39.9 (98.7)	40.4 (100.0)	0.162
Amikacin	0 (0.0)	4 (2.8)	0.242	1.3	0 (0.0)	1.1 (2.8)	0.240
Kanamycin	0 (0.0)	2 (1.4)	0.170	1.3	0 (0.0)	0.2 (0.6)	0.110
Streptomycin	5 (6.3)	21 (14.9)	0.281	1.3	3.2 (7.9)	4.8 (12.0)	0.137
ALIS	3 (3.8)	4 (2.8)	0.054	1.3	0.9 (2.3)	1.0 (2.4)	0.006
Later-generation fluoroquinolone	3 (3.8)	5 (3.5)	0.013	1.3	1.6 (3.9)	1.8 (4.5)	0.030

Categorical variables are presented as number (%), and continuous variables are presented as mean ± standard deviation. Missing values are presented as percentages of the total population.

Abbreviations: AFB, acid-fast bacillus; ALIS, amikacin liposome inhalation suspension; BMI, body mass index; C-NB, cavitary nodular bronchiectatic; COPD, chronic obstructive pulmonary disease; EMB, ethambutol, FC, fibrocavitary; *M. avium*, *Mycobacterium avium*; *M. intracellulare*, *Mycobacterium intracellulare*; NC-NB, noncavitary nodular bronchiectatic; SMD, standardized mean difference; TB, tuberculosis; UC, unclassifiable.

^a^The dose of EMB in the low-dose EMB group was <12.5 mg/kg/d.

^b^The dose of EMB in the high-dose EMB group was ≥12.5 mg/kg/d.

Table 2 summarizes the clinical outcomes in the original dataset before multiple imputation. None of the 79 patients (0%) in the low-dose group and 7 of the 144 patients (4.9%) in the high-dose group developed ethambutol-induced optic neuropathy. Seven of the 79 patients (8.9%) in the low-dose group and 15 of the 144 patients (10.4%) in the high-dose group had failure of negative culture conversion. However, 13 of the 79 patients (16.7%) in the low-dose group and 48 of the 144 patients (34.8%) in the high-dose group were unable to produce sputum so negative culture conversion could not be assessed. None of the 79 patients (0%) in the low-dose group and 2 of the 144 patients (1.4%) in the high-dose group developed macrolide resistance.

**Table 2.** Clinical Outcomes in the Original Dataset Before Imputation

Variables	Low-Dose EMB^a^ n = 79 n (%)	High-dose EMB^b^ n = 144 n (%)	*P*	Missing (%)
Primary endpoint	…	…		…
Diagnosis of optic neuropathy	0 (0)	7 (4.9)	0.053	0.0
Secondary endpoints	…	…		…
Failure of negative culture conversion	7 (8.9)	15 (10.4)	0.817	0.0
Development of macrolide resistance	0 (0)	2 (1.4)	0.540	0.0

EMB, ethambutol.

^a^The dose of EMB in the low-dose EMB group was <12.5 mg/kg/d.

^b^The dose of EMB in the high-dose EMB group was ≥12.5 mg/kg/d.

The clinical outcomes based on datasets generated from the data imputed using the bootstrap method are presented in Table 3. In the unadjusted cohort, the incidence of ethambutol-induced optic neuropathy was 0% (95% CI: 0.0%–0.0%) in the low-dose group and 4.8% (95% CI: 1.5%–8.7%) in the high-dose group, a risk difference of −4.8% (95% CI: −8.7% to −1.5%). The proportion of patients with failure of negative culture conversion was 8.5% (95% CI: 2.9%–15.3%) in the low-dose group and 10.3% (95% CI: 5.6%–15.8%) in the high-dose group, a risk difference of −1.8% (95% CI: −9.5% to 6.5%). The incidence of macrolide resistance was 0% (95% CI: 0.0%–0.0%) in the low-dose group and 1.4% (95% CI: 0.0%–3.6%) in the high-dose group, a risk difference of −1.4% (95% CI: −3.6% to 0.0%). In the adjusted cohort, the low-dose group had a significantly lower incidence of optic neuropathy compared with that of the high-dose group, with a risk difference of −17.1% (95% CI: −32.9% to −5.4%). The risk difference for failure of negative culture conversion was −20.0% (95% CI: −45.6% to 2.5%) and the incidence of macrolide resistance was −4.9% (95% CI: −13.5% to .0%), and did not differ significantly between the groups.

**Table 3.** Summary of Absolute Risk on Clinical Outcomes of the Unadjusted and Adjusted Cohort in the Data Set Generated From the Imputed Data Using the Bootstrap Method

Variables	Unadjusted Cohort			Adjusted Cohort		
	Low-Dose EMB^a^	High-Dose EMB^b^	Absolute risk difference	Low-Dose EMB^a^	High-Dose EMB^b^	Absolute risk Difference
Primary endpoint	…	…	…	…	…	…
Diagnosis of optic neuropathy (%)	0 (0.0–0.0)	4.8 (1.5–8.7)	–4.8 (–8.7 to –1.5)	0 (0.0–0.0)	17.1 (5.4–32.9)	−17.1 (−32.9 to −5.4)
Secondary endpoints	…	…	…	…	…	…
Failure of negative culture conversion (%)	8.5 (2.9–15.3)	10.3 (5.6–15.8)	−1.8 (−9.5 to 6.5)	17.2 (5.7–30.7)	37.3 (19.9–59.6)	−20.0 (−45.6 to 2.5)
Development of macrolide resistance (%)	0 (0.0–0.0)	1.4 (0.0–3.6)	−1.4 (−3.6 to 0.0)	0 (0.0–0.0)	4.9 (0.0–13.5)	−4.9 (−13.5 to 0.0)

Risk differences are presented as the risk in the low-dose EMB group minus that in the high-dose EMB group.

The numbers in the parentheses in the absolute risk and risk difference indicates the 95% CI.

EMB, ethambutol.

^a^The dose of EMB in the low-dose EMB group was <12.5 mg/kg/d.

^b^The dose of EMB in the high-dose EMB group was ≥12.5 mg/kg/d.

Subsequently, we performed a sensitivity analysis including patients with a treatment duration of 5 years or less (78 in the low-dose group and 134 in the high-dose group). In this subgroup analysis, treatment duration was well-balanced between the groups (mean: 516 vs 514 days; SMD: 0.006), and excellent balance was achieved for all other variables after adjustment (all SMDs <0.1; Supplementary Table 1). Optic neuropathy occurred in 0 (0.0%) patients in the low-dose group compared with 7 (5.2%) patients in the high-dose group (Supplementary Table 2). Based on the bootstrap-generated datasets, the analysis in the adjusted group showed a significantly lower risk of optic neuropathy in the low-dose group (risk difference: −18.1%; 95% CI: −35.1% to −5.5%), with no significant differences in secondary endpoints (Supplementary Table 3).

## DISCUSSION

In this single-center retrospective cohort study in patients with MAC-PD, we evaluated the association between low-dose ethambutol therapy and optic neuropathy. After adjusting for patient background using overlap weighting based on propensity scores, the low-dose ethambutol group had a significantly lower incidence of optic neuropathy. Additionally, the proportion of patients with failure of negative culture conversion did not differ significantly between the 2 groups. Moreover, the incidence of macrolide resistance was lower in the low-dose group than in the high-dose group, although this difference was not statistically significant.

According to current international guidelines, multidrug treatment with a combination of at least 3 antibiotics (including a macrolide and ethambutol) is recommended for treating MAC-PD [1]. In patients with the NC-NB type, intermittent therapy (a 3-times per week regimen) is recommended because of the comparable rate of negative conversion and favorable tolerability compared with that of daily therapy [1]. In addition, intermittent therapy has been shown to have fewer interruptions in ethambutol and a lower incidence of optic neuropathy compared with that of daily therapy [17, 27, 28]. However, daily therapy is recommended for patients with cavitary or severe bronchiectasis owing to the lack of evidence of the effectiveness of intermittent therapy for these types [1]. Therefore, further studies are needed to determine the appropriate daily dose of ethambutol to reduce the risk of optic neuropathy. Although the incidence of optic neuropathy has been reported to increase in a dose-dependent manner [29, 30], the appropriate dose cutoff to balance the occurrence of optic neuropathy and the effectiveness of treatment has not been determined. Our study builds on 2 previous studies from Japan [12, 13], and demonstrates that not only was the incidence of optic neuropathy lower in the low-dose ethambutol group, but also that the effectiveness of treatment was similar in patients treated with low-dose and high-dose ethambutol, after adjustment for differences in patient characteristics. Therefore, the evidence for low-dose ethambutol treatment, particularly with regard to the reduction in the incidence of optic neuropathy, has become more robust.

A major strength of this study is that we adjusted for differences in patient characteristics between the low-dose and high-dose ethambutol groups, including risk factors for ethambutol-induced optic neuropathy and prognostic factors for NTM-PD. The main reported risk factors for ethambutol-induced optic neuropathy other than the ethambutol dose are older age and kidney dysfunction [14, 31]. Multiple studies have identified older age as a risk factor [14–16, 31]. A recent large-scale, single-center retrospective study of risk factors for ethambutol-induced optic neuropathy conducted in Thailand reported that age over 60 years was an independent risk factor for optic neuropathy [15]. Similarly, a recent study of ethambutol users in Korea, which used a nationwide database, showed that advanced age was a risk factor for optic atrophy and visual impairment in the multivariate analyses [16]. This study found that older patients were more vulnerable than younger patients to severe structural and visual outcomes. Another similar study of ethambutol users in Taiwan, which also used a nationwide database, reported similar findings [31]. In addition, kidney dysfunction has also been reported as an important risk factor for ethambutol-associated optic neuropathy [31, 32]. Because ethambutol is rapidly absorbed and primarily excreted via the kidneys, its half-life is significantly prolonged in individuals with advanced kidney dysfunction, resulting in elevated blood concentrations and an increased risk of ocular toxicity [32, 33]; therefore, careful dose adjustment based on creatinine clearance is essential [32]. When evaluating the incidence of ethambutol-associated optic neuropathy, advanced age and kidney dysfunction are important confounding factors. In this study, the low-dose group was older and had a higher creatinine clearance in the unadjusted analysis, whereas these variables were well balanced between groups in the adjusted analysis. Other known risk factors, such as hypertension [31] and ophthalmic diseases [12], were also accounted for in the adjustment of patient characteristics.

The potential influence of ethambutol treatment duration on optic neuropathy risk is an important consideration. While the time-to-onset of optic neuropathy after ethambutol initiation varies—from within 6 months to over a year [10, 14, 34]—evidence for treatment duration as an independent risk factor remains inconsistent [15, 31, 32, 35]. In contrast, a recent large-scale study using a Korean nationwide database identified cumulative dose as a significant independent risk factor [16]. While treatment duration has not been definitively confirmed as an independent risk factor, its possible contribution to the development of optic neuropathy cannot be disregarded. In our cohort, the mean duration of ethambutol administration was longer in the high-dose group than in the low-dose group (642 vs 534 days; SMD = 0.198). To minimize the impact of treatment duration on our results, we performed a sensitivity analysis restricted to patients treated with ethambutol for 5 years or less. This analysis yielded results consistent with our primary findings, further supporting the robustness of the association between a lower daily dose and reduced risk of optic neuropathy.

This study also benefited from being conducted in a tertiary care hospital with ophthalmologists, allowing for systematic assessment of any visual disturbances. The diagnostic procedures including tests of visual acuity, field of vision, color perception, and central flicker were consistently applied, facilitating accurate diagnosis of ethambutol-related optic neuropathy.

The effectiveness of treatment, which was evaluated as a secondary endpoint, showed that failure of negative culture conversion did not differ significantly between the high- and low-dose groups in both the unadjusted and adjusted analyses. These results suggest that lowering the ethambutol dose does not compromise the overall effectiveness of treatment. Notably, none of the patients in the lower-dose group developed macrolide resistance, consistent with previous reports that low-dose ethambutol therapy does not increase the risk of developing macrolide resistance [12, 13].

These findings suggest that clinicians may consider reducing the ethambutol dose as a strategy to improve treatment tolerability by reducing the risk of optic neuropathy in patients requiring daily therapy for MAC-PD. Moreover, our results further support this approach by demonstrating that ethambutol doses below 12.5 mg/kg may be sufficient to maintain the effectiveness of treatment and reduce the risk of macrolide resistance. Additionally, low-dose therapy may enhance long-term safety, especially given the frequent recurrence of MAC-PD [21, 36]. By lowering the daily dose and thereby reducing cumulative exposure, clinicians could effectively mitigate toxicity risks for patients requiring repeated therapeutic courses over time. However, owing to limited data on the minimum effective dose of ethambutol, any dose reduction should be undertaken cautiously.

This study has some limitations. First, external validity may be limited owing to the retrospective single-center design. However, the relatively large sample size and adjustment for confounding using propensity score-based overlap weighting strengthens its internal validity. Second, although the analysis adjusted for patient characteristics using propensity score analysis, the possibility of residual confounding cannot be ruled out. Third, missing data were imputed using bootstrap-based variance estimation. Although this method is statistically robust if the data are missing at random, this assumption may not fully reflect the complexity of real-world clinical data. Fourth, in this study, a large portion of patients were unable to expectorate sputum, limiting the evaluation of the effectiveness of treatment using standard microbiological endpoints such as culture conversion and microbiological cure. This might also have affected the assessment of macrolide resistance. To address this limitation, we used failure of negative culture conversion as an alternative outcome indicator, consistent with a previous study [12]. Fifth, more than 60% of our study population had NC-NB type disease, which typically has a more favorable prognosis than C-NB or FC type disease [21]. Consequently, the external validity of our findings may be limited for patients with more severe and refractory phenotypes. This is particularly relevant to the interpretation of our secondary endpoints. Sixth, the patients in our study had relatively low BMIs (mean: 20.7 kg/m^2^ in the low-dose group and 18.7 kg/m^2^ in the high-dose group), reflecting the lean or cachectic physique characteristic commonly observed in MAC-PD. Consequently, our findings may have limited generalizability to patients with higher body weights or obesity. In such individuals, higher absolute daily doses of ethambutol could lead to a higher cumulative dose—a known risk factor for optic neuropathy [16]—and thus the safety and optimal dosing strategy (including the choice of adjusted, ideal, or total body weight for dose calculation) in this population remain to be determined. Seventh, while our sensitivity analysis restricted to patients treated for 5 years or less supported the primary findings, these results should be interpreted with caution. Since the onset of optic neuropathy necessitates immediate ethambutol discontinuation, treatment duration is not a truly independent variable but is partially determined by the clinical outcome. Consequently, this analysis may be subject to selection bias due to conditioning on a post-treatment variable. Eighth, although the ethambutol shortage was of limited duration, this resulted in temporary regimen modifications that might have affected the outcomes. Finally, our study focused exclusively on patients with pulmonary MAC disease; individuals with disseminated or extrapulmonary MAC disease were not included in this cohort. Therefore, whether a low-dose ethambutol regimen is effective and safe for these specific manifestations remains uncertain. Future multicenter, prospective cohort studies with standardized microbiological monitoring and complete data capture are warranted to confirm the robustness of these findings and to further refine the optimal dosing strategy for ethambutol in clinical practice.

## CONCLUSIONS

In conclusion, after adjusting for patient characteristics, low-dose ethambutol therapy was associated with a significantly lower incidence of optic neuropathy without compromising treatment outcomes. These findings suggest that dose reduction may help to lower the risk of ethambutol-induced optic neuropathy. Further large-scale prospective cohort studies are warranted to confirm these findings and to establish the optimal dosing strategy.

## Supplementary Material

ofag127_Supplementary_Data

## Supplementary Data

Supplementary materials are available at *Open Forum Infectious Diseases* online. Consisting of data provided by the authors to benefit the reader, the posted materials are not copyedited and are the sole responsibility of the authors, so questions or comments should be addressed to the corresponding author.
