# Supplementary material for: Association Between Low-Dose Ethambutol Therapy and Optic Neuropathy in Mycobacterium avium Complex Pulmonary Disease: A Retrospective Cohort Study Using Propensity Score Analysis
Source: Open Forum Infect Dis. 2026 Mar 3;13(3):ofag127. doi: 10.1093/ofid/ofag127 (PMC12989744; doi:10.1093/ofid/ofag127)
Supplement: ofag127_Supplementary_Data [file ofag127_supplementary_data.docx]

**Association between Low-dose Ethambutol Therapy and Optic Neuropathy in *Mycobacterium avium* Complex Pulmonary Disease: A Retrospective Cohort Study Using Propensity Score Analysis**

Supplementary Data

Supplementary Table S1 Baseline clinical characteristics of the unadjusted and adjusted cohort before imputation in the subgroup with an ethambutol treatment duration of five years or less

| Variables | Unadjusted cohort | | | | Adjusted cohort | | |
| --- | --- | --- | --- | --- | --- | --- | --- |
|  | Low-dose EMB^a^  n = 78 | High-dose EMB^b^  n = 134 | SMD | Missing  (%) | Low-dose EMB^a^  n = 37.91 | High-dose EMB^b^  n = 37.91 | SMD |
| EMB dose (mg/kg/day) | 10.71 (± 1.34) | 15.57 (± 2.74) | 2.252 | 0.9 | 10.77 (± 1.34) | 14.97 (± 2.10) | 2.389 |
| EMB dose (mg/day) | 543 (± 102) | 705 (± 136) | 1.344 | 0.0 | 529 (± 87) | 720 (± 126) | 1.773 |
| Duration of EMB administration (days) | 516.4 (± 375.6) | 514.1 (± 415.5) | 0.006 | 0.0 | 527.3 (± 370.3) | 526.2 (± 417.6) | 0.003 |
| Age (years) | 70.5 (± 8.9) | 68.8 (± 9.4) | 0.184 | 0.0 | 70.0 (± 9.0) | 70.0 (± 8.8) | < 0.001 |
| Female | 64 (82.1) | 101 (75.4) | 0.164 | 0.0 | 30.0 (79.1) | 30.0 (79.1) | < 0.001 |
| BMI (kg/m^2^) | 20.8 (± 3.5) | 18.7 (± 3.0) | 0.650 | 10.7 | 19.9 (± 2.9) | 19.9 (± 2.9) | < 0.001 |
| Smoking history | 18 (23.4) | 33 (25.4) | 0.047 | 2.3 | 9.2 (24.8) | 7.4 (20.1) | 0.113 |
| Previous history of TB treatment | 4 (5.1) | 3 (2.2) | 0.154 | 0.0 | 1.7 (4.5) | 1.0 (2.7) | 0.097 |
| Underlying disease |  |  |  |  |  |  |  |
| Ophthalmic disorders | 9 (11.5) | 21 (15.7) | 0.121 | 0.0 | 5.7 (15.0) | 5.7 (15.0) | < 0.001 |
| Hypertension | 21 (26.9) | 34 (25.8) | 0.026 | 0.9 | 9.0 (23.7) | 9.0 (23.7) | < 0.001 |
| Diabetes mellitus | 7 (9.0) | 16 (11.9) | 0.097 | 0.0 | 3.0 (7.9) | 4.5 (11.8) | 0.131 |
| COPD | 5 (6.4) | 7 (5.2) | 0.051 | 0.0 | 2.8 (7.3) | 1.5 (4.0) | 0.145 |
| Bronchial asthma | 2 (2.6) | 2 (1.5) | 0.074 | 0.9 | 0.8 (2.2) | 0.9 (2.4) | 0.013 |
| Interstitial pneumonia | 2 (2.6) | 13 (9.8) | 0.305 | 0.9 | 0.7 (1.8) | 3.8 (10.0) | 0.356 |
| Aspergillosis | 1 (1.3) | 3 (2.3) | 0.075 | 0.9 | 0.8 (2.0) | 1.2 (3.1) | 0.065 |
| Lung cancer | 2 (2.6) | 5 (3.8) | 0.068 | 1.4 | 0.7 (1.8) | 1.1 (2.9) | 0.073 |
| Species |  |  |  |  |  |  |  |
| *M. avium* | 53 (67.9) | 64 (49.2) | 0.387 | 1.9 | 23.5 (61.9) | 21.7 (57.4) | 0.092 |
| *M. intracellulare* | 30 (38.5) | 72 (55.4) | 0.344 | 1.9 | 17.3 (45.6) | 17.3 (45.6) | < 0.001 |
| Positive AFB smear | 32 (41.0) | 46 (35.9) | 0.105 | 2.8 | 15.0 (39.6) | 15.0 (39.6) | < 0.001 |
| Radiological disease type |  |  | 0.370 | 10.3 |  |  | < 0.001 |
| NC-NB form | 47 (61.0) | 77 (68.1) |  |  | 25.1 (67.9) | 25.1 (67.9) |  |
| C-NB form | 21 (27.3) | 19 (16.8) |  |  | 7.8 (21.2) | 7.8 (21.2) |  |
| FC form | 6 (7.8) | 16 (14.2) |  |  | 3.4 (9.1) | 3.4 (9.1) |  |
| UC form | 3 (3.9) | 1 (0.9) |  |  | 0.7 (1.8) | 0.7 (1.8) |  |
| Blood biochemistry |  |  |  |  |  |  |  |
| Albumin (g/dL) | 3.96 (±0.52) | 3.77 (±0.65) | 0.333 | 5.6 | 3.97 (±0.48) | 3.77 (±0.70) | 0.323 |
| Creatinine (mg/dL) | 0.64 (±0.14) | 0.71 (±0.50) | 0.203 | 0.0 | 0.65 (±0.14) | 0.65 (±0.17) | 0.036 |
| Creatinine clearance (mL/min) | 70.49 (±22.26) | 62.03 (±20.10) | 0.399 | 0.9 | 67.28 (±17.54) | 67.28 (±20.32) | < 0.001 |
| C-reactive protein (mg/dL) | 0.40 (±0.69) | 1.11 (±2.41) | 0.393 | 5.6 | 0.47 (±0.78) | 1.01 (±2.52) | 0.287 |
| Drug use |  |  |  |  |  |  |  |
| Macrolide | 78 (100.0) | 134 (100.0) |  | 0.0 | 37.9 (100.0) | 37.9 (100.0) |  |
| Clarithromycin | 37 (47.4) | 111 (82.8) |  | 0.0 | 18.9 (49.9) | 28.7 (75.7) |  |
| Azithromycin | 41 (52.6) | 23 (17.2) | 0.800 | 0.0 | 19.0 (50.1) | 9.2 (24.3) | 0.555 |
| Rifampicin | 77 (98.7) | 134 (100.0) | 0.161 | 0.0 | 37.4 (98.7) | 37.9 (100.0) | 0.163 |
| Amikacin | 0 (0.0) | 4 (3.1) | 0.251 | 1.4 | 0 (0.0) | 1.1 (3.0) | 0.249 |
| Kanamycin | 0 (0.0) | 2 (1.5) | 0.176 | 1.4 | 0 (0.0) | 0.3 (0.8) | 0.126 |
| Streptomycin | 4 (5.1) | 19 (14.5) | 0.319 | 1.4 | 2.6 (6.8) | 3.9 (10.4) | 0.127 |
| ALIS | 3 (3.8) | 4 (3.1) | 0.043 | 1.4 | 0.8 (2.2) | 1.1 (2.9) | 0.043 |
| Later-generation fluoroquinolone | 3 (3.8) | 5 (3.8) | 0.002 | 1.4 | 1.4 (3.8) | 1.9 (5.1) | 0.063 |

^a^The dose of EMB in the low-dose EMB group was <12.5 mg/kg/day. ^b^The dose of EMB in the high-dose EMB group was ≥12.5 mg/kg/day.

Categorical variables are presented as number (%), and continuous variables are presented as mean ± standard deviation. Missing values are presented as percentages of the total population.

Abbreviations: AFB, acid-fast bacillus; ALIS, amikacin liposome inhalation suspension; BMI, body mass index; C-NB, cavitary nodular bronchiectatic; COPD, chronic obstructive pulmonary disease; EMB, ethambutol, FC, fibrocavitary; *M. avium*, *Mycobacterium avium*; *M. intracellulare*, *Mycobacterium intracellulare*; NC-NB, non-cavitary nodular bronchiectatic; SMD, standardized mean difference; TB, tuberculosis; UC, unclassifiable.

Supplementary Table S2 Clinical outcomes in the original dataset before imputation in the subgroup with an ethambutol treatment duration of five years or less

| Variables | Low-dose EMB^a^  n = 78  n (%) | High-dose EMB^b^  n = 134  n (%) | *P* | Missing  (%) |
| --- | --- | --- | --- | --- |
| **Primary endpoint** |  |  |  |  |
| Diagnosis of optic neuropathy | 0 (0) | 7 (5.2) | 0.049 | 0.0 |
| **Secondary endpoints** |  |  |  |  |
| Failure of negative culture conversion | 7 (9.0) | 13 (9.7) | 1.000 | 0.0 |
| Development of macrolide resistance | 0 (0) | 1 (0.7) | 1.000 | 0.0 |

^a^The dose of EMB in the low-dose EMB group was <12.5 mg/kg/day. ^b^The dose of EMB in the high-dose EMB group was ≥12.5 mg/kg/day.

EMB, ethambutol

Supplementary Table S3 Summary of absolute risk on clinical outcomes of the unadjusted and adjusted cohort in the subgroup with an ethambutol treatment duration of five years or less in the data set generated from the imputed data using the bootstrap method

| Variables | Unadjusted cohort | | | Adjusted cohort | | |
| --- | --- | --- | --- | --- | --- | --- |
|  | Low-dose  EMB^a^ | High-dose   EMB^b^ | Absolute risk difference | Low-dose  EMB^a^ | High-dose   EMB^b^ | Absolute risk difference |
| **Primary endpoint** |  |  |  |  |  |  |
| Diagnosis of optic neuropathy, % | 0 (0.0–0.0) | 5.1 (1.6–9.3) | –5.1 (–9.3 to –1.6) | 0 (0.0–0.0) | 18.1 (5.5–35.1) | –18.1 (–35.1 to –5.5) |
| **Secondary endpoints** |  |  |  |  |  |  |
| Failure of negative culture conversion, % | 8.6 (2.9–15.5) | 9.6 (5.0–14.9) | –0.9 (–8.7 to 7.3) | 18.3 (6.1–33.1) | 34.2 (17.2–55.9) | –15.6 (–41.1 to 7.4) |
| Development of macrolide resistance, % | 0 (0.0–0.0) | 0.7 (0.0–2.4) | –0.7 (–2.4 to 0.0) | 0 (0.0–0.0) | 2.6 (0.0–9.1) | –2.6 (–9.1 to 0.0) |

^a^The dose of EMB in the low-dose EMB group was <12.5 mg/kg/day. ^b^The dose of EMB in the high-dose EMB group was ≥12.5 mg/kg/day.

Risk differences are presented as the risk in the low-dose EMB group minus that in the high-dose EMB group.

The numbers in the parentheses in the absolute risk and risk difference indicates the 95% confidence interval.

EMB, ethambutol
